# Unmasking the invisible enemy: A case report of metagenomics-guided diagnosis and treatment of neonatal septic meningitis caused by *Corynebacterium aurimucosum* in a preterm infant with neonatal lupus erythematosus

**DOI:** 10.1097/MD.0000000000035968

**Published:** 2024-02-16

**Authors:** Qing Xia, Meiqun Gu, Yu Xu, Haoke Sang, Wenhua Lin, Yajun Wang, Kai Liu

**Affiliations:** a Kunming Children’s Hospital, Pulmonary and Critical Care Medicine, Kunming, China; b The Affiliated Hospital of Kunming University of Science and Technology, The First People’s Hospital of Yunnan Province, Kunming, PR China; c Chinese People’s Liberation Army Logistic Support Army No. 920 Hospital, Kunming, China.

**Keywords:** *Corynebacterium aurimucosum*, neonatal lupus erythematosus, septic meningitis

## Abstract

**Rationale:**

Neonatal septic meningitis is a serious condition that can be caused by various pathogens, including *Corynebacterium aurimucosum*, a rare and opportunistic bacterium. We reports a case of infectious meningitis in a premature infant with neonatal lupus erythematosus caused by *C aurimucosum*. The purpose of this study is to explore the occurrence of meningitis caused by *C aurimucosum* in preterm infants with neonatal lupus erythematosus. We found that early diagnosis and treatment are crucial for this type of meningitis, especially for infants with impaired immunity or mothers receiving immunosuppressive therapy. This bacterium is rare in clinical practice, but it needs to be taken seriously.

**Patient concerns:**

The infant was born to a mother with systemic lupus erythematosus who had a history of long-term immunosuppressive therapy. The infant presented with preterm birth, purplish-red skin, fever, and widespread scarlet dermatitis. He also had positive anti-Ro/SSA and anti-La/SSB antibodies.

**Diagnosis:**

The infant was diagnosed with neonatal lupus erythematosus based on clinical and serological features. A lumbar puncture revealed septic meningitis with high levels of total nucleated cells, protein, and Pan’s test in the CSF. The macrogenic examination identified *C aurimucosum* as the causative agent. The culture of the mother’s vaginal secretion also revealed the same bacterium.

**Interventions:**

The infant was treated with anti-infective therapy with ceftriaxone, ampicillin, vancomycin, and meropenem. He also received prednisone and gammaglobulin infusion for neonatal lupus erythematosus.

**Outcomes:**

The infant’s temperature returned to normal, and his general condition and responsiveness improved. The CSF cytology and biochemistry normalized, and the culture was negative. The cranial MRI examination showed no abnormalities. The red rash disappeared, and the follow-ups after discharge revealed no complications.

**Lessons:**

This case highlights the importance of early diagnosis and treatment of neonatal septic meningitis caused by *C aurimucosum*, especially in infants with immunocompromised conditions or maternal history of immunosuppressive therapy. *C aurimucosum* should not be overlooked as a potential pathogen in neonatal septic meningitis.

## 1. Introduction

Neonatal septic meningitis is one of the most common acute and critical illnesses affecting newborns; however, the absence of specific signs and symptoms makes early diagnosis challenging. The immune system of neonates is not fully developed, and the blood–brain barrier protection is relatively feeble; therefore, bacteria can easily invade the body, cause infectious diseases such as sepsis, and readily cross the blood–brain barrier and cause septic meningitis. *Corynebacterium aurimucosum* is a highly uncommon pathogen that causes meningitis. Besides Corynebacterium diphtheriae, Corynebacterium is less pathogenic and seldom causes sepsis. Corynebacterium is often overlooked and misdiagnosed when identified as a contaminant. A case of neonatal lupus erythematosus and septic meningitis caused by *C aurimucosum* infection was recently admitted to our neonatal unit.

## 2. Case presentation

### 2.1. Chief complaints

The infant was admitted due to ``preterm birth,’’ presenting with purplish-red skin and extremities, rapid breathing, and perioral cyanosis.

### 2.2. History of present illness

**Figure 1.**
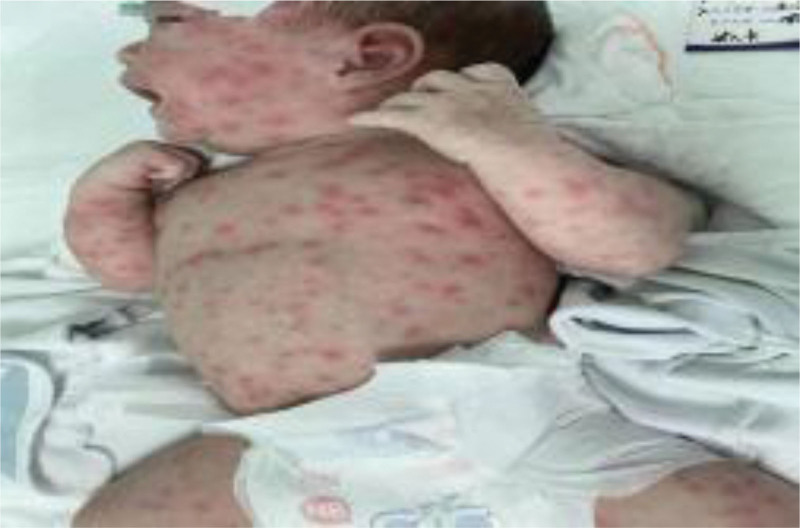
Prominent facial and head rash. The rash is red and covers most of the patient’s face and head.

The male infant was born preterm in May 2023 in the Obstetrics Department of the First People’s Hospital of Yunnan Province, with a birth weight of 2125 g. The Apgar scores were 9, 9, and 9 at 1, 5, and 10 minutes after birth. During the third trimester, the amniotic fluid was observed to be cloudy. Immediately after birth, the infant’s skin was purplish-red. However, the baby’s skin transitioned to a red and moist appearance after airway clearance and warming. Subsequently, the infant developed a fever and widespread scarlet dermatitis, as shown in Figure 1.

### 2.3. History of past illness

The mother was diagnosed with SLE and was under regular follow-up, maintaining a well-controlled condition. She conceived in August 2022. During her pregnancy, she was administered prednisone 15 mg daily, cyclosporine 25 mg twice daily, hydroxychloroquine 0.2 mg twice daily, and calcium carbonate D3 0.6 mg daily. In mid-December 2022, she contracted COVID-19, leading to paralysis and muscle pain in the extremities and joints. During her hospitalization, her ANAs tested positive for dsDNA and other autoantibodies. She was diagnosed with lupus activity due to limb and joint muscle discomfort. Two urine cultures revealed *Escherichia coli*, and she was recommended cefuroxime for the infection. A sputum culture detected multi-drug resistant Citrobacter fluorides. However, as she did not exhibit symptoms like cough or fever, it was considered bacterial colonization, and no anti-infective treatment was given. Before her discharge, her medications were adjusted to prednisone 45 mg orally once daily, cyclosporine 100 mg twice daily, and hydroxychloroquine 0.2 mg twice daily.

### 2.4. Physical examination

The baby weighed 2.12 kg and was in a generally poor condition. He had rapid and rhythmic breathing, perioral cyanosis, moaning, and foaming at the mouth. Breath sounds were coarse in both lungs without discernible dry or wet rales. Heart, abdomen, and nervous system examinations did not reveal any abnormalities.

### 2.5. Laboratory examinations

**Figure 2.**
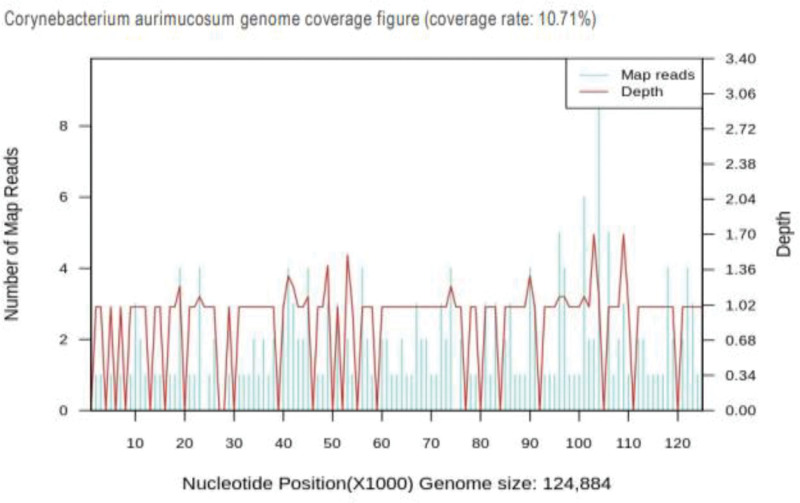
*Corynebacterium aurimucosum* genome coverage figure. The figure shows the depth of coverage across the genome of the *Corynebacterium aurimucosum* isolate.

During the turbulent period, routine blood tests repeatedly showed white blood cell count (10.03–17.81) × 10^9^/L, hemoglobin 175–248 g/L, platelets 130–162 × 10^9^/L, and ultrasensitive C-reactive protein at 0.5 mg/L. ANAs revealed positive results for ANA, RNP, SS-A, and SS-B. The cerebrospinal fluid (CSF) appeared yellowish-red slightly turbid, with PAN test ++, total nucleated cells at 1073 × 10^9^/L, glucose at 0.7 mmol/L, and protein quantification at 2714 mg/L. Subsequent lumbar punctures indicated whole nucleated cells of 1632 × 10^9^/L and then 3 × 10^9^/L. Glucose levels adjusted from 0.3 mmol/L to 1 mmol/L, and protein levels changed from 1650 mg/L to 862 mg/L. The macrogenic CSF examination indicated *C aurimucosum*, as shown in Figure 2.

### 2.6. Imaging examinations

**Figure 3.**
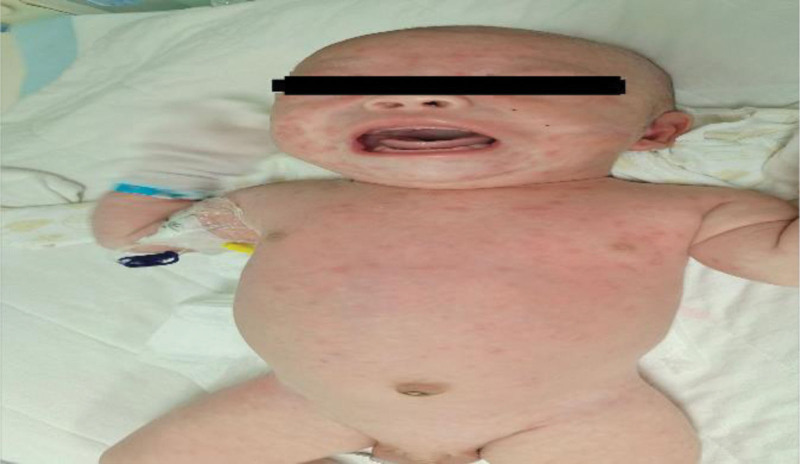
Disappearance of the red rash. The rash has faded and is no longer visible on the patient’s skin.

A computed tomography scan of the infant was conducted, revealing no abnormalities. The cranial MRI, depicted in Figure 3, also showed no anomalies.

## 3. Final diagnosis

Neonatal lupus erythematosus (NLE).

Septic meningitis due to *C aurimucosum*.

## 4. Treatment

Upon hospital admission, the infant was placed in an incubator and was supported with a noninvasive ventilator. Given the onset of fever and the appearance of the scarlet dermatitis, he was initially treated with ampicillin. After the septic meningitis diagnosis, the treatment regimen was expanded to include ceftriaxone and ampicillin. As the infant continued to experience recurrent fevers, another lumbar puncture was performed, leading to the addition of prednisone at 2.5mg PO QD and gammaglobulin infusion to the treatment. The persistence of the fever led to another treatment adjustment, replacing the antibiotics with a combination of vancomycin and meropenem, based on the sensitivity profile of *C aurimucosum*.

## 5. Outcome and follow-up

### 5.1. Outcome

Posttreatment, the infant’s fever subsided, and there was a significant improvement in his overall condition and responsiveness. The parameters of the CSF normalized after the entire course of anti-infective treatment.

### 5.2. Follow-up

Subsequent cranial MRI examinations revealed no abnormalities. Follow-ups at 1 week and 1 month post-discharge demonstrated that the infant was on a positive recovery trajectory with no new exceptions noted.

### 5.3. Ethical considerations

Considering the utilization of data from a vulnerable/minor population in this case presentation, it is hereby stated that written informed consent was secured from the legal guardian/next of kin for the patient in this specific age group.

## 6. Discussion

NLE is a rare acquired autoimmune disease affecting the fetus and neonate,^[1]^ first described by McCuistion and Schoch^[2]^ in 1954, and frequently manifesting with congenital heart block and/or annular erythema of the skin. The following are the diagnostic criteria for neonatal lupus erythematosus: Congenital heart blocks in a newborn with positive maternal and/or neonatal anti-Ro/SSA and/or anti-La/SSB antibodies; the presence of skin lesions associated with NLE as determined by a dermatologist and/or histopathologist in a newborn with positive maternal and/or neonatal anti-Ro/SSA and/or anti-La/SSB antibodies. Positive antibodies. NLE is diagnosed when any of the above conditions are met. This child is diagnosed with neonatal lupus erythematosus based on generalized dermatitis on the face and head, the presence of anti-SAA antibodies, and the long-term SLE history of the mother.

Neonatal septic meningitis is one of the most prevalent acute and life-threatening conditions affecting newborns. It is a postnatal infectious central nervous system disease predominantly caused by meningitis. According to studies,^[3]^ *Escherichia coli* and *Streptococcus pneumonia* were the most prevalent bacteria in cases of meningitis in neonatal intensive care unit wards in Canada between January 2010 and December 2016. Neonates are at risk for septic meningitis due to an immature immune system, weak neutrophil, and monocyte function, inadequate secretion of complement and antimicrobial proteins and peptides, and postnatal attenuation of maternal antibodies.^[4,5]^ The nonspecific clinical symptoms of neonatal septic meningitis include fever, hypothermia, lethargy, irritability, vomiting, and respiratory changes. Neonatal septic meningitis is typically transmitted through prenatal, perinatal, and postnatal routes. The prenatal course is the transmission of infection from the mother to the fetus through the placenta before delivery; the intrapartum practice occurs when the membranes rupture prematurely, and the risk of prolonged delivery; and the postnatal path is the invasion of pathogens into the body due to factors such as a newborn’s compromised immune system and blood–brain barrier function.^[6]^ Due to newborns’ limited immune and blood–brain barrier function, the postnatal route is utilized. In this article, the child is a preterm newborn with a poor immune status, a mother with systemic lupus erythematosus, a history of long-term hormone and immunosuppressant administration before delivery, and a mother who was hospitalized at 24 weeks of gestation with sputum culture of Citrobacter fulminant (multi-drug resistant), a widespread and conditional pathogen.^[7]^ The application of adrenal glucocorticosteroids and immunosuppressive drugs further decreased the immune function of the patient’s organism, damaged the phagocytosis and physiological defensive barrier function of the cells, and disrupted the ecological balance of the host’s normal flora for an extended period, leaving the host vulnerable to infection by other opportunistic pathogens. Without significant abnormalities in inflammatory indexes, a complete lumbar puncture suggested septic meningitis based on the child’s spontaneous delivery and subsequent fever. A lumbar puncture was performed on the child, which revealed septic meningitis. Simultaneously, the macro genetic examination of the neonate, and the culture of the mother’s vaginal secretion all revealed the presence of bacillus aurimucosums.

After a patient recovers, Corynebacterium, also known as “diphtheria” or “rod” bacteria, is frequently identified as a contaminant. However, accumulating evidence indicates that many of these strains are conditionally pathogenic.^[8]^ Only Corynebacterium diphtheriae was once considered an infectious agent. However, routine immunization against these pathogens is now available, and the pathogenesis of Corynebacterium diphtheriae is uncommon. However, non-DPTB have garnered more attention in recent years as opportunistic human pathogens.^[8,9]^ Several case reports have confirmed that Corynebacterium can be highly drug-resistant and pathogenic in recent years due to the enhancement of medical care, the increase in immunocompromised patients, and the extensive use of novel testing techniques.^[10–12]^ The bacterium is exceptionally resistant to antibiotics. Corynebacterium can cause tissue and systemic infections, including, among others, bacteremia, respiratory tract infections, breast infections, endocarditis, and osteomyelitis.^[13]^ The precise identification of *C aurimucosum* and the investigation of other species is a challenging endeavor. Nevertheless, it is necessary to identify the reliable source of infection and administer the appropriate treatment.^[14]^ *C aurimucosum* was described as a novel species of the genus Corynebacterium in 2002.^[15]^ There is a need to report clinically significant isolates from various samples that can be used to determine the spectrum of potential infections caused by this pathogen. This strain has been isolated from multiple clinical samples; it can cause cutaneous infections^[16]^ or erythema secondary to vaginal colonization and may be associated with spontaneous abortion.^[3,17]^ Following urethroplasty, a urethral stricture infection was documented in 2012.^[18]^ In 2012, a condition of the urinary tract induced by urethral stricture after urethroplasty was reported. Meningitis influenced by *Corynebacterium rotundum* is uncommon in China. Meningitis caused by Corynebacterium spp. has been reported relatively more frequently abroad. A patient with malignant lymphoma whose meninges were infiltrated by tumor cells ultimately passed away due to severe meningitis complicated by Corynebacterium.^[19]^ Corynebacterium is highly resistant, with a high rate of penicillin resistance, moderate resistance to ciprofloxacin, cotrimoxazole, imipenem, and meropenem, and a low rate of erythromycin resistance.^[20]^ It was found to be susceptible to both vancomycin and linezolid.^[21]^ The disease was not severe. Due to the infant’s critical condition and the delayed drug sensitivity test results, vancomycin was chosen as the anti-infective treatment. After an entire course of anti-infective therapy, the child’s clinical symptoms completely vanished, and the CSF routine, biochemistry, and culture returned to normal.

The use of metagenomics in neonatal medicine has shown promising results in improving the diagnosis and treatment of neonatal infections. Metagenomic sequencing can provide rapid and accurate identification of pathogens, including rare and opportunistic bacteria such as *C aurimucosum*, allowing for timely and personalized treatment. Metagenomics can also reveal the diversity and function of the neonatal microbiome, which plays a crucial role in the development and health of the newborn. Metagenomics can help to elucidate the factors that influence the establishment and maturation of the neonatal microbiome, such as maternal health, mode of delivery, feeding type, antibiotic exposure, and environmental factors. Metagenomics can also help to identify the associations between the neonatal microbiome and various neonatal diseases, such as sepsis, necrotizing enterocolitis, bronchopulmonary dysplasia, and allergic disorders. Further research is needed to fully explore the potential of metagenomics in neonatal medicine and to translate the findings into clinical practice.^[22–24]^

The summarized limitations and challenges from the article: this study focuses on a rare case of neonatal lupus erythematosus complicated by *C aurimucosum* meningitis. The findings may not be generalizable to other types of neonatal meningitis or different patient populations. Identifying and diagnosing *C aurimucosum* can be challenging due to its rarity and opportunistic nature. Additionally, recognizing and treating such infections in neonates, whose immune systems are not fully developed, can be particularly difficult. Although the patient recovered well with antimicrobial and lupus treatment, *C aurimucosum*’s resistance to multiple antibiotics poses treatment challenges. Further research is needed to validate these findings and determine the exact role of *C aurimucosum* in neonatal meningitis. Exploring the potential of metagenomics in neonatal medicine and translating these discoveries into clinical practice is essential. The study also lacks whole-genome sequencing and comparative analysis of *C aurimucosum* strains, further investigation into bacterial transmission pathways, and dynamic monitoring of the infant’s microbiome during treatment.

## 7. Conclusion

this case highlights the importance of early diagnosis and treatment of neonatal septic meningitis caused by *C aurimucosum*, especially in infants with immunocompromised conditions or maternal history of immunosuppressive therapy. The use of metagenomics can provide rapid and accurate identification of pathogens, allowing for timely and personalized treatment. *C aurimucosum* should not be overlooked as a potential pathogen in neonatal septic meningitis.

## Acknowledgments

We owe our thanks to Kai Liu for his work on revising this manuscript.

## Author contributions

**Conceptualization:** Qing Xia, Meiqun Gu, Yu Xu, Haoke Sang, Wenhua Lin, Yajun Wang, Kai Liu.

**Resources:** Meiqun Gu.

**Software:** Yu Xu.

**Supervision:** Kai Liu.

**Writing – review & editing:** Wenhua Lin.

## Supplementary Material




