# Supplementary material for: Unmasking the invisible enemy: A case report of metagenomics-guided diagnosis and treatment of neonatal septic meningitis caused by Corynebacterium aurimucosum in a preterm infant with neonatal lupus erythematosus
Source: Medicine (Baltimore). 2024 Feb 16;103(7):e35968. doi: 10.1097/MD.0000000000035968 (PMC10869058; doi:10.1097/MD.0000000000035968)
Supplement: Supplementary file 1 [file medi-103-e35968-s001.pdf]

云南省第一人民医院

发表病例报告知情同意书

尊敬的患者家属，您好：

经住院后有关检查，当前考虑您的孩子诊断为：粘金色棒状杆菌脑膜炎，脑脊液培养及药敏试验提示粘金色棒状杆菌对万古霉素敏感，经过足量及足疗程抗感染治疗后，您的孩子症状及脑脊液复查已经恢复正常。目前粘金色棒状杆菌导致新生儿化脓性脑膜炎在国际上暂无报道，属于罕见病例，我们希望发布有关内容，加强医师对棒状杆菌的致病性的认识及该病治疗手段的认识。发布的有关文章会隐去患儿的个人信息，但会交代整个发病过程及治疗转归。假定您赞同，可签订此知情同意书。

同意发表

杨男

医师：夏青

日期：2023.06.01.

扫描全能王
3亿人都在用的扫描App
