# Supplementary material for: Unmasking the invisible enemy: A case report of metagenomics-guided diagnosis and treatment of neonatal septic meningitis caused by Corynebacterium aurimucosum in a preterm infant with neonatal lupus erythematosus
Source: Medicine (Baltimore). 2024 Feb 16;103(7):e35968. doi: 10.1097/MD.0000000000035968 (PMC10869058; doi:10.1097/MD.0000000000035968)
Supplement: Supplementary file 2 [file medi-103-e35968-s002.pdf]

云南省第一人民医院
儿科特殊用药知情同意书

姓名：杨琴之子　　性别：男性　　出生日期：2023年05月01日(0岁
科室：儿科　　床号：075　　住院号：8487437

尊敬的患者、患者的法定监护人或授权委托人：
根据患者目前的病情，患者具有该治疗适应症，医师特向您详细介绍和说明如下内容：该项治疗必要性、目的性、医疗风险、并发症和可能存在的医疗意外等情况，帮助您了解相关知识、作出选择。

医师告知

【目前诊断】1.新生儿化脓性脑膜炎2.母体系统性红斑狼疮新生儿3.先天性肺炎4.早产儿(孕期等于或大于28整周，但小于32整周)5.低出生体重儿(1500-2499克)6.母体年状腺功能减退新生儿7.高危儿8.新生儿病理性黄疸9.新生儿红斑狼疮

【拟行治疗名称】万古霉素药物治疗

【治疗原因和目的】

【治疗风险和注意事项】

(1)本治疗介绍：医生已告知我孩子有　　_______，需要进行万古霉素药物治疗。

(2)本治疗的风险包括但不限于：医生告知我万古霉素治疗可能发生的一些风险，有些不常见的风险可能没有在此列出，具体的治疗方式根据不同病人的情况有所不同，医生告诉我可与孩子的主管医生讨论有关孩子治疗的具体内容，如果我有特殊的问题可与孩子的主管医生讨论。

1.我理解任何治疗都存在风险。

2.我理解任何所用药物都可能产生副作用，包括轻度的恶心、皮疹等症状到严重的过敏性休克，甚至危及生命。

3.我理解此治疗可能发生的风险：

(1)休克，过敏样症状；
(2)急性肾功能不全，间质性肾炎；
(3)多种血细胞减少；
(4)皮肤粘膜综合征(Stevens-Johnson综合征)，中毒性表皮坏死症；
(5)第8脑神经损伤症状；
(6)伪膜性大肠炎；肝功能损害，黄疸等。
(7)该药物价格昂贵，使用后可能无效；
(8)其他目前无法预料的风险和并发症。

4.我理解治疗后如果不遵医嘱，可能影响治疗效果。

(3)本治疗注意事项：

【预期的治疗效果】控制感染

【替代检查方案及其风险和效果】

1.利奈唑胺：价格昂贵，可能疗效欠佳；
2.青霉素：可能耐药。

【拒绝治疗可能产生的后果】感染加重

【患者自身存在的高危因素】

合并以上高危因素的情况下，患者在检查中或检查后可能随时出现病情变化、加重，甚至死亡。

1.根据患者的病情，患者需要进行上述医疗措施。该措施是一种有效的诊疗手段，一般来说是安全的，但由于该措施具有创伤性和风险性，因此医师不能向患者保证措施的效果。

扫描全能王
3亿人都在用的扫描App

云南省第一人民医院

儿科特殊用药知情同意书

姓名：杨琴之子　　性别：男性　　出生日期：2023年05月01日(0岁)

科室：儿科　　床号：075　　住院号：8487437

医师声明

一旦发生上述风险或其他意外情况，医师将从维护患者利益出发积极采取应对措施。

2.对病变器官、组织、标本及影像等资料，医疗机构可进行相应合理处置，包括检测检验、病理检查、教学和科学研究等，终末标本按医疗废物规范处置。

3.我已经尽量以患者所能了解之方式，解释该措施的相关信息，特别是下列事项：

(1)实施该措施的原因、目的、风险；

(2)并发症及可能处理方式；

(3)不实施该措施可能发生的后果及其他可替代诊疗方式；

(4)如另有关于此措施的相关说明情况，我已如实告知。

医师签名：[illegible]　　签字时间：2023年05月13日

云南省第一人民医院 儿科

患方声明及签字

1.医师已向我解释，并且我已经了解实施该医疗措施的必要性、步骤、风险、成功率之相关信息。

2.医师已向我解释，并且我已经了解选择其他医疗措施之风险。

3.医师已向我解释，并且我已经了解该医疗措施的风险和不实施该医疗措施的风险。

4.针对我孩子的情况，我能够向医师提出问题和疑虑，并已获得说明。

5.我了解该医疗措施可能是目前最适当的选择，但是其仍然存在风险且无法保证一定能够达到预期目的。

6.我已经向医师如实地介绍了病史，尤其是与本医疗措施有关的病史。

7.紧急情况处置授权：本人明白除了医生告知的危险以外，医疗方案实施中有可能出现其他危险或者预想不到的情况，在此我也授权医师，在遇到预料之外的紧急、危险情况时，从考虑患者利益角度出发，按照医学常规予以处置。

8.医师已经给予我充足时间，询问有关拟实施医疗措施的问题，并给予答复(如无疑问，请填写无)：

基于上述声明，我 同意 (填同意或不同意)对患者实施该项医疗措施。

患者签字：　　(手印)　签字时间：　　年　月　日　时　分

如果患者无法签署告知书，请其法定监护人、授权委托人在此签名：

患者法定监护人/授权委托人签字：杨琴　　(手印)

与患者关系：母子

签字时间：2023 年 5 月 13 日 10 时 20 分

是否为《授权委托书》的受托人或是《特别声明人》的声明人☑是□否

备注

如果患者、患者的法定监护人或授权委托人拒绝签字，请医师在此栏中说明：

说明人：

时间：　　年　月　日　时　分

CS 扫描全能王
3亿人都在用的扫描App
